# A Ciliary View of the Immunological Synapse

**DOI:** 10.3390/cells8080789

**Published:** 2019-07-29

**Authors:** Chiara Cassioli, Cosima T. Baldari

**Affiliations:** Department of Life Sciences, University of Siena, 53100 Siena, Italy

**Keywords:** primary cilium, immunological synapse, ciliary proteins, extraciliary functions, T lymphocytes

## Abstract

The primary cilium has gone from being a vestigial organelle to a crucial signaling hub of growing interest given the association between a group of human disorders, collectively known as ciliopathies, and defects in its structure or function. In recent years many ciliogenesis proteins have been observed at extraciliary sites in cells and likely perform cilium-independent functions ranging from regulation of the cytoskeleton to vesicular trafficking. Perhaps the most striking example is the non-ciliated T lymphocyte, in which components of the ciliary machinery are repurposed for the assembly and function of the immunological synapse even in the absence of a primary cilium. Furthermore, the specialization traits described at the immunological synapse are similar to those seen in the primary cilium. Here, we review common regulators and features shared by the immunological synapse and the primary cilium that document the remarkable homology between these structures.

## 1. Introduction

Activation of naïve T cells and execution of effector functions by terminally differentiated T cells involves the specific recognition of a peptide-loaded major histocompatibility complex (pMHC) by the T cell receptor (TCR) on the surface of an antigen presenting cell (APC) or a target cell, respectively. Antigen-independent interactions of co-stimulatory receptors and adhesion molecules with complementary ligands are also required. Signaling triggers an extensive rearrangement of receptors and cytoskeletal changes that culminate in the establishment of a transient cell polarity with the assembly of a specialized structure, known as the immunological synapse (IS) [1,2,3,4]. Over the last decade, seminal studies have suggested a homology between the IS and the primary cilium. Additionally, an unexpected role in IS formation is played by an increasing number of “ciliary” proteins that were described for their localization and function in the primary cilium and are now identified as active participants in IS-related functions in the non-ciliated T cells. These findings corroborate the notion that, despite the absence of a primary cilium, the T cell has maintained the expression of many proteins involved in ciliogenesis and repurposed them to build the IS. Therefore, the usage of the “ciliary” label for proteins that localize outside the cilium and perform extraciliary functions needs to be reconsidered. 

Here we provide an overview of the striking similarities between the IS and the primary cilium, both at structural and functional levels. We further present a detailed comparison of the IS with the primary cilium, discussing differences and similarities in establishing cell polarity, actin and microtubule cytoskeleton remodeling, centrosome positioning, polarized vesicular trafficking and phosphoinositide signaling, on which both IS assembly and ciliogenesis crucially depend. 

## 2. The Immunological Synapse and the Primary Cilium at a Glance: More Similarities Than Differences

The concept of the IS dates back to the early 1980s, when TCR-dependent Ca^2+^ signaling was linked to cell adhesion and cytokine secretion [5]. However, this structure has garnered the interest of scientists only after the development of outstanding models and imaging strategies to visualize it. In the canonical configuration, the IS resembles a “bull’s eye” with receptors and adhesion molecules distributed in three concentric regions that Kupfer defined supramolecular activation clusters (SMAC) [6,7]. The TCR and the costimulatory receptor CD28 are clustered in the central SMAC (cSMAC), surrounded by a peripheral ring (pSMAC) of LFA-1 and a distal ring (dSMAC) of glycoproteins with bulky ectodomains, such as the sialophorin CD43 and the tyrosine phosphatase CD45 [8,9]. 

Long before, the primary cilium was described in mammalian cells by Zimmerman, and given its name in the 1960s by Sorokin, who noticed that it first appears during the development of the central nervous system [10]. Similar to motile cilia, the primary cilium is a microtubule-based organelle that extends from a modified centriole, known as the basal body. However, the primary cilium differs from motile cilia in that it is a single organelle emerging from the surface of almost all cell types and, with few exceptions (i.e., olfactory and nodal cilia), it contains a 9 + 0 axoneme lacking the central microtubule pair and dynein arms. The significance of this small organelle remained elusive for a century, until the association between defects in ciliary growth or function and human diseases brought it to the limelight [11,12].

At a first glance, the IS and the primary cilium appear as completely different structures. Moreover, although under specific experimental conditions (i.e., serum deprivation or depletion of the negative regulator of ciliogenesis centriolar coiled-coil protein 110 kDa) immortalized T and B cells have been reported to form a rudimentary cilium [13], hematopoietic cells do not have a primary cilium and as such would be expected to lack the proteins that localize and function within this organelle. Surprisingly, a more detailed analysis has revealed that both the IS and the primary cilium share striking specialization traits and functions that we will discuss in this review.

### 2.1. Similarities in the Architectural Framework of the IS and the Primary Cilium

**Figure 1:**
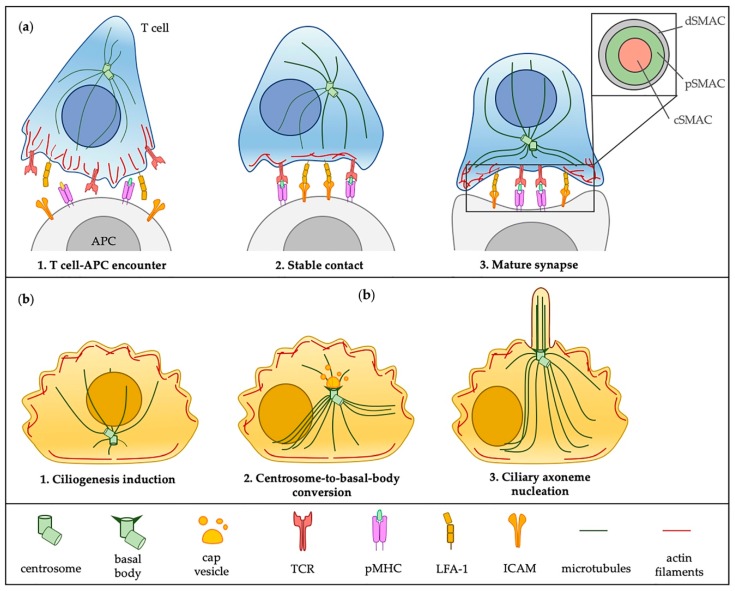
Schematic representation of critical steps in immunological synapse (IS) assembly and ciliogenesis. Both IS assembly and ciliogenesis are inducible processes that are initiated in response to external stimuli or triggering events. The encounter of an antigen presenting cell (APC) bearing a cognate peptide-loaded major histocompatibility complex (pMHC) initiates the formation of a stable IS in the T cell (**a**). At variance, ciliogenesis is activated in vitro by a variety of stressful conditions (e.g., serum and nutrient starvation, ultraviolet light radiation), which generally inhibit cell division (**b**). In the T cell the centrosome moves toward the synapse as a consequence of early T cell receptor (TCR) signaling events (**a**) and, at this location, sets the stage for polarized vesicular trafficking. Cilium assembly crucially depends on centrosome-to-basal-body conversion that consists in the polarization and subsequent docking of the mother centriole to the plasma membrane, where it nucleates the ciliary axoneme. During ciliogenesis, centrosome repositioning is associated with the Rab11-Rab8 dependent generation and expansion of a cap vesicle above the mother centriole (**b**). At the IS newly polymerized actin filaments contribute to the initial clustering of TCRs in the central supramolecular activation clusters (cSMAC). Following polarization of the centrosome, actin retracts to the distal SMAC (dSMAC) to form a ring, which surrounds the peripheral SMAC (pSMAC) enriched in LFA-1 (**a**). A redistribution of actin in contractile bundles at the ventral side and a cortical network at the dorsal side helps to break cell symmetry and promotes centrosome migration during ciliogenesis (**b**). In both structures the docking phase of the centrosome is concomitant with a local clearance of cortical actin.

The assembly of both the IS and the primary cilium (Figure 1) requires a transient break of cell symmetry and the translocation of the centrosome close to the plasma membrane. In ciliated cells the centrosome translocates to the surface, whereupon the mother centriole anchors to the plasma membrane through its distal appendages and forms the basal body to template the primary cilium. Similarly, in immune cells the centrosome advances to the cell periphery and there is evidence of mother centriole docking to the synaptic membrane of cytotoxic lymphocytes (CTLs) [14]. However, a difference in the timing of centrosome orientation has been observed. In CTLs the centrosome rapidly polarizes and retracts, allowing them to sequentially kill multiple targets [15], while the migration of the centrosome to the apical pole during ciliogenesis requires a longer time [16] and its retraction is concomitant with cell cycle re-entry.

Interestingly, while the cilium is a well-defined appendage that projects from the cell surface to the extracellular space, a small bump reminiscent of a cilium has been observed by electron microscopy at the lytic IS that forms between a CTL and a target cell [17]. Another similarity between the IS and the primary cilium is the localization of the Golgi apparatus, a key organelle in the orchestration of vesicular trafficking. In 1985 Poole described a non-random orientation of the Golgi apparatus with the *trans*-Golgi network (TGN) always pointing toward the primary cilium [18]. Since the Golgi apparatus and the recycling compartment are closely associated with the centrosome, both polarize to the IS membrane after centrosome repositioning [19]. Moreover, the tight relationship of the Golgi apparatus with the primary cilium has been underscored by the identification of a dual localization for the ciliary protein intraflagellar transport (IFT) 20, which was found both at the basal body and the Golgi apparatus through binding to the golgin GMAP-210 in ciliated cells [20]. Similarly, IFT20 expressed in the non-ciliated T cell shows an extensive colocalization with both the centrosome and the Golgi compartment and moves just beneath the IS in T cell:APC conjugates [21]. In addition, GMAP-210 has recently been identified as a molecular partner of IFT20 in T cells [22], confirming its association with the Golgi apparatus even in the absence of a primary cilium. 

### 2.2. Functions Shared by the IS and the Primary Cilium

The comparison between the IS and the primary cilium extends beyond their architectural framework. Both structures have been extensively characterized as signaling platforms and show a local clustering of cholesterol/sphingolipid-enriched domains as well as membrane receptors and signaling mediators. At the IS, a bidirectional flow of both chemical and physical signals between a naïve T cell and the APC occurs in the form of receptor:ligand interactions and mechanical forces across the cell–cell interface. This ensures the exchange of information between immune cells resulting in a finely tuned modulation of the adaptive immune response [3,23,24,25]. More challenging was the discovery of a signaling function for the primary cilium. Although it was considered as an organelle that had lost its motility, the primary cilium has been clearly demonstrated to function as the “cell’s antenna” [26] and plays a key role in a wide range of processes by transducing a variety of signaling pathways in vertebrates [27]. 

In addition to and in many instances related to signaling, the IS and the primary cilium are sites of massive vesicular trafficking. The polarization of the centrosome and the Golgi apparatus are determinant for the establishment of a directional transport of vesicles toward and away from the IS that as a result acts as a focal point for both endocytosis and exocytosis. While the cSMAC was initially considered the synaptic domain that allows for sustained signaling, it is now clear that TCR microclusters (TCR-MCs) signal during their centripetal movement toward the IS center and have become signaling-incompetent by the time they reach the cSMAC [28,29]. There ligand-engaged TCRs undergo receptor-mediated endocytosis and are routed to alternative fates (i.e., recycling, degradation, signaling from endosomes or incorporation into ectosomes) [30]. The active removal of receptors from the cSMAC is not only a means to terminate signaling, but is also exploited to facilitate the replacement of exhausted TCRs with signaling-competent receptors associated with recycling endosomes. Indeed, the intracellular pool of TCRs is mobilized to the IS during the long-lasting contact with the APC, allowing signaling to proceed for hours. In addition to the TCR, vesicles containing other recycling receptors (e.g., the transferrin receptor, the chemokine receptor CXCR4) [31,32,33] as well as membrane-associated signaling components (e.g., the lymphocyte-specific protein tyrosine kinase Lck, the transmembrane adaptor linker for activation of T cells LAT) [34,35] are delivered to the synaptic membrane. This exocytic function is maximized in helper (Th) and cytotoxic T cells, which form immune synapses with B cells and target cells, respectively, for the focalized secretion of cytokines and costimulatory ligands (Th cells) or lytic granules and apoptosis-inducing molecules (CTLs) into the “sealed” space of the synaptic cleft [2].

Similar to the IS, cilia assembly and maintenance rely on vesicular trafficking of protein and lipid components from the TGN and recycling endosomes to the primary cilium. However, some membrane-bound components exploit an alternative route and reach the cilium from the plasma membrane through lateral diffusion [36]. Recently, an invagination of the plasma membrane that forms at the base of the cilium, called the ciliary pocket, has emerged as a central hub for cilia-associated vesicular trafficking [37], where both endocytic and exocytic events contribute to regulate signaling at the primary cilium. In this regard, a growing body of literature has provided evidence for the negative regulation of multiple signaling pathways via receptor internalization at the ciliary pocket (e.g., Sonic hedgehog receptor Patched 1, transforming growth factor β, G protein-coupled receptors (GPCRs), Notch) [38]. 

Lastly, in addition to their pivotal role as signal receivers, both the IS and the primary cilium are active players in producing outwards signals. A large number of extracellular vesicles (EVs), including ectosomes and exosomes, are focally released from the IS and the cilium into the extracellular milieu [39,40]. These vesicles function as carriers in cell–cell communication and the response of the target cell varies depending on the specific cargo (i.e., signaling components, DNA, mRNA and microRNAs, enzymes). Although some studies carried out on *Caenorhabditis elegans* have attempted to shed light on ciliary-derived EVs [41,42], their function is still largely unknown. The release of vesicles by ciliated sensory neurons of *C. elegans* into the surrounding environment has led to the hypothesis of a role for EVs in interindividual communication and mating behavior. However, shedding vesicles from the ciliary tip to the extracellular space have been reported by Nachury and colleagues. In this case, vesicle release appears a strategy that ciliated cells employ for clearing active receptors, when their retrieval from the cilium to the cell body fails [43]. New insights into exocytic traffic at the IS have revealed that TCR-containing ectosomes are released into the synaptic cleft between a CD4^+^ T cell and a cognate B cell upon antigen receptor triggering. Choudhury et al. have shown that centrally clustered TCRs escape in part from lysosome-mediated degradation and are instead sorted by TSG101, a protein of the endosomal sorting complex required for transport (ESCRT)-I, into ectosomes that bud from the synaptic membrane. These vesicles are taken up by B cells bearing cognate pMHC on the surface and promote sustained signaling required for B cell maturation [44]. In addition to the TCR, death receptor ligands (i.e., FasL and APO2L/TRAIL) and microRNAs [45,46] have been identified as cargoes of synaptic vesicles. Furthermore, Torralba et al. have recently shown that T cells prime dendritic cells through the focal release of exosomes enriched in genomic and mitochondrial DNA. The transfer of DNA-containing EVs between immune cells induces a responsive state in dendritic cells, allowing them to promptly become activated in subsequent viral infections [25]. Hence synaptic vesicles may be expected to mediate a cell-cell crosstalk aimed at modulating the immune response both under physiological and pathological conditions, however further work is required to elucidate the mechanisms responsible for EV generation and function. 

## 3. A “Ciliary” View of the Immunological Synapse

As discussed above, the assembly and function of both the IS and the primary cilium depend on the cytoskeleton dynamics and polarized vesicular trafficking. Intriguingly, there is now compelling evidence that many ciliary proteins localize at extraciliary sites and carry out multiple functions, beyond ciliogenesis. The growing list of extraciliary functions ranges from orientation of the mitotic spindle, to cell cycle regulation, DNA damage response, phosphoinositide metabolism, centrosomal positioning, cytoskeleton remodeling and orchestration of vesicular trafficking. In a very thought-provoking review, Hua and Ferland have recently proposed the hypothesis that ciliary proteins could be general regulators of cell polarity and that, as a result of this function, they orchestrate the development not only of the primary cilium but also of other polarized structures, including the IS in T cells, and the growth cones and dendritic spines in neuronal cells [47]. Here we will detail how the cytoskeleton, vesicular trafficking, phospholipids and polarity proteins participate in the assembly and function of both the IS and the primary cilium, highlighting the similarities between these structures. 

### 3.1. Cytoskeleton Regulates Assembly and Function of the IS and the Primary Cilium 

The formation of the primary cilium and the IS entails profound changes on the cell surface that reflect a dramatic reorganization of the cytoskeleton at the intracellular side. The cytoskeleton acts as a master organizer and structural scaffold of the cell and consists, among other things, of microtubules and actin filaments. Both these polymers are characterized by a dynamic instability and switch between growing and shrinking phases. The polymerization-depolymerization dynamics of microtubules and actin filaments generate forces that not only shape the IS and the primary cilium, but also affect their function through molecular motors that directionally move cargo along microtubules or actin filaments. A number of regulatory proteins and post-translational modifications of tubulin contribute to the microtubule-based functions at the primary cilium and the IS. Furthermore, a role for septins, a novel component of the cytoskeleton, has now emerged in ciliogenesis and their synaptic localization could predict potential implications for IS assembly as well. 

#### 3.1.1. Pushing or Pulling: How the Centrosome Moves toward the Apical Membrane

The primary cilium and the IS share the property of being organized above the centrosome, which untethers from the nucleus and repositions just beneath the plasma membrane [10,19]. At this location, the centrosome acts as a microtubule organizing center (MTOC) and orchestrates the microtubule-dependent transport of vesicles, allowing for ciliary elongation and function in ciliated cells, and for the sustained delivery of endosome-associated receptors and signaling mediators to the IS in T cells. The centrosome and the microtubular network are also essential for the polarization of intracellular organelles and compartments (i.e., the Golgi apparatus, the endo-lysosomal system and mitochondria). Although microtubules actively grow from the centrosome once it polarizes to the IS [48], a unique property of the centrosome of ciliated cells that is not shared by T cells is to act as a template for microtubule elongation to form the axoneme [49].

An early event in the intracellular pathway of ciliogenesis is the centriole-to-basal-body conversion. When ciliogenesis is induced, the mother centriole recruits vesicles positive for the recycling endosome-associated GTPase Rab11, which supply membranes for the generation of a cap structure, known as the ciliary vesicle. The recruitment and activation of Rab8 is required for the expansion of this vesicle, which moves in association with the centrosome toward the apical surface and eventually fuses with the plasma membrane [50]. At the apical pole, the mother centriole’s distal appendages anchor the basal body to the plasma membrane and axoneme nucleation initiates. Similar to ciliated cells, a capping vesicle has been recently described on the top of one of the centrioles in Jurkat T cells [51], even though its biogenesis has not been investigated yet. Nevertheless, it is noteworthy that in T cells centrosome polarization is a consequence of TCR activation, rather than a triggering event in IS assembly. Moreover, this process occurs faster compared to ciliated cells. RPE1 ciliated cells expressing EGFP-centrin-1 showed that the apical translocation of the centrosome initiates 2 h after serum deprivation and completes within 8 h [16], while in lymphocytes centrosome repositioning was observed within 5 min of TCR stimulation [52]. Previous studies had confirmed the role of TCR signaling in centrosome polarization by demonstrating that mediators of TCR signaling (i.e., Lck/Fyn, ZAP-70, SLP-76 and LAT) [53,54,55,56,57] as well as the diacylglycerol (DAG)-dependent activation of PKC [58,59] are required for centrosome localization to the IS, while calcium mobilization [59] and the integrin LFA-1 [60] are dispensable. 

Pushing forces generated by microtubule polymerization and pulling forces involving molecular motors are the mechanical forces that drive centrosome repositioning. The contribution of these forces may vary depending on cell types or movement distance and may also account for the difference in translocation timing. In ciliated cells, Pitaval et al. have described an increased density of microtubules surrounding the centrosome, which cluster in a large bundle and generate pushing forces as a major contribution to basal body propulsion toward the plasma membrane [16]. This observation does not exclude a minor participation of minus end-directed motors at a later stage, since an increased presence of the dynein-interacting proteins p150^Glued^ and NuMA at the apical cortex was also reported [16,61,62]. Microtubule polymerization and depolymerization also occur during centrosome repositioning in T cells [48,52,63]. Treatment of Jurkat T cells with low concentrations of microtubule-targeting drugs, namely taxol (blocking microtubule depolymerization) or nocodazole (blocking microtubule polymerization), has been shown to affect the synaptic positioning of the centrosome [52]. Consistent with these observations, phosphorylation of the tubulin-sequestering protein stathmin 1 by Erk in activated T cells regulates centrosome polarization by increasing free cytoplasmic tubulin [64]. Similarly, ciliated cells depleted of stathmin show an increased frequency of microtubule bundles pointed toward the basal pole in the presence of serum [16]. Moreover, casein kinase 1δ has been shown to activate the microtubule plus-end binding protein EB1, thus promoting microtubule growth during centrosome translocation to the IS [65]. Of note, both casein kinase 1δ and EB1 have been implicated also in ciliogenesis [66,67]. Another factor that contributes to centrosome repositioning in T cells is post-translational modifications of tubulin that influence biophysical parameters of microtubules as well as the recruitment of microtubule-binding proteins. Acetylation and detyrosination are unique features of stable microtubules and are accordingly detected at high levels at the ciliary axoneme [68]. Interestingly, emerging evidence suggests that centrosome polarization in T cells requires a stabilized microtubule network. Indeed, the deacetylase HDAC6, which has been involved in cilia disassembly and mechanosensitivity [69,70,71], also controls centrosome translocation in both CD4^+^ and CD8^+^ T cells [72,73]. Moreover, activated T cells show high levels of detyrosinated microtubules and two independent studies have identified inverted formin-2 and microtubule associated protein 4 as key players in this process [74,75].

In addition to the pushing forces generated by microtubule dynamics and post-translational modifications of tubulin, pulling forces are a central factor in centrosomal repositioning to the IS in T cells. The minus-end directed motor dynein has long been implicated in centrosome polarization [60,76], consistent with its rapid recruitment to the IS after DAG generation [59]. However, the static view of dynein trapped at the dSMAC through the binding with adhesion and degranulation adaptor proteins has changed following the observation that dynein centripetally moves together with TCR-MCs toward the cSMAC [77]. Additionally, live cell imaging has shown a microtubule end-on capture-shrinkage mechanism that is affected by the inhibition of both microtubule depolymerization and dynein [52,78]. Recently, Lim et al. have posited that the microtubule plus-end tracking protein CLIP-170 contributes to dynein localization at the IS. Based on their results, the authors proposed that in stimulated cells a CLIP-170-dependent transport of dynein toward the microtubule plus-ends is decreased in favor of a more stable interaction of dynein with membrane proteins in the center of the synapse, where it generates a pulling force that allows the centrosome to localize close to the synaptic membrane [78]. Importantly, molecular motor coupling to microtubules is regulated by post-translational modifications of the latter. While kinesin preferentially interacts with acetylated microtubules, dynactin facilitates initial binding of dynein to tyrosinated microtubules through its p150^Glued^ subunit and EB proteins. Once initiated, dynein movement can proceed on detyrosinated microtubules without requiring dynactin [79]. Since detyrosinated microtubules are enriched around the centrosome and further increase after TCR engagement [74], this would influence the initial interaction of dynein with microtubules leading to major consequences on centrosome repositioning to the IS and intracellular traffic. 

Hence a hallmark of the nascent IS and primary cilium is centrosome repositioning, a process that relies on a combination of cytosolic and cortical forces. A full understanding of how the centrosome is driven to a new cellular location during the assembly of the IS and the primary cilium requires further studies and still represents a challenging question. 

#### 3.1.2. Dropping Anchor at the Plasma Membrane

Once it has reached the apical pole, the centrosome establishes a physical contact with the plasma membrane and some efforts have been made to define the components required for this process. Through a small interfering RNA screen for proteins implicated in ciliogenesis, the distal appendage protein CEP164 has been implicated in primary cilium formation and later in centrosome migration [80]. In addition, CEP83 and OFD2 regulate the function of distal appendages and as such contribute to primary cilia formation [81,82]. Similar to what happens in early ciliogenesis, Stinchombe et al. have observed that in CTLs the mother centriole docks directly to the synaptic membrane through its distal appendages and that depletion of the distal appendage protein CEP83 results in an impaired secretion activity [14]. However, there is still no evidence of a physical contact between the centrosome and the synaptic membrane in CD4^+^ T cells. Surprisingly, treatment of Jurkat cells with the dynein inhibitor ciliobrevin D impairs the docking phase of the polarized centrosome [52], suggesting that the search and capture of microtubule plus-ends mechanism at the cell cortex may indirectly link the centrosome to the plasma membrane, even without the participation of distal appendages. 

#### 3.1.3. The Actin Cytoskeleton Contributes to Ciliogenesis

The primary cilium has long been considered as an actin-deficient organelle. Nevertheless, immunogold labelling of mature mouse photoreceptors has shown the presence of actin in the distal portion of these specialized cilia [83]. A proteomic analysis of the ciliary membrane has confirmed actin-binding proteins as ciliary components [84]. Additionally, recent studies have identified a novel role for the actin cytoskeleton in the regulation of ciliogenesis and cilia length. The assembly of an ezrin-rich actin cortical network on the apical surface facilitates ciliogenesis, while an increased density of cytoplasmic actin stress fibers inhibits cilia formation [85]. Conversely, disruption of actin polymerization, either by depletion of action-related proteins 2/3 (e.g., Arp2/3) or cytocalasin D treatment, improves ciliary assembly [85,86]. 

The distribution of cytoplasmic and cortical actin also influences basal body positioning and docking. At the onset of ciliogenesis, myosin II (MyoII) activity mediates the contraction of ventral actin filaments that leads to a lateral displacement of the nucleus, thus favoring centrosome movement [16]. This is consistent with the finding that the loss of actin-binding proteins, such as meckelin and nesprin-2, impairs centrosome positioning by redistributing actin into cytoplasmic stress fibers [87]. At a later stage, the actin-based motor MyoVa is transported by dynein to the pericentrosomal region, where it mediates the recruitment of vesicles to the distal appendages of the mother centriole, thereby ensuring cilia assembly [88]. Surprisingly, a co-regulation of cortical actin and tubulin, which are both substrates of the deacetylase HDAC6, has been reported [89], suggesting an involvement of actin in ciliary disassembly as well. In support of this notion, MyoVa was found to accumulate in the cilium during ciliary resorption [84]. Moreover, actin polymerization within the ciliary compartment has been implicated in an unusual strategy of cilium loss involving the detachment of the ciliary tip. This phenomenon, known as “ciliary decapitation”, was reported in IMCD3, RPE1 and NIH3T3 cells grown under nutrient-rich conditions [90]. Interestingly, Phua et al. have recently demonstrated that an early feature in ciliary disassembly is the removal of the phosphatase INPP5E from the ciliary membrane, which results in an enhanced local concentration of phosphatidylinositol 4,5-bipshosphate (PtdIns(4,5)P_2_). This phosphoinositide influences the ciliary localization of actin regulators, such as cofilin-1, fascin and the small GTPase K-Ras, that could cooperate in actin polymerization, followed by actin-mediated pinching of the ciliary membrane tip and the generation of a truncated cilium. Although ciliary decapitation is a means for the rapid loss of the upper part of the cilium, it does not account for the complete disassembly of this organelle, since tubulin was not found in the fragments of decapitated cilia. 

Lastly, actin regulates Hedgehog (Hh) signaling in primary mouse dermal cells [91]. Furthermore, the release of ectosomes containing activated signaling molecules from the tip of cilia is an actin-dependent process [43]. 

#### 3.1.4. The Actin Cytoskeleton Controls Mechanical Communication at the IS

At variance with ciliogenesis, actin dynamics has been long established as a central process in IS assembly. Actin undergoes a profound remodeling during IS assembly with an initial polymerization of new actin filaments at the synapse, followed by clearance from the center of the contact area. This is concomitant with the formation of the ring structure of the pSMAC that stabilizes the T cell:APC interaction by preventing the diffusion of molecules that accumulate at the cSMAC [92]. Actin retraction is instrumental for polarized microtubule-driven vesicular trafficking to and from the plasma membrane on which T cell activation and effector function crucially depend, as clearly exemplified by CTLs. Namely, Griffiths and colleagues have demonstrated that actin depletion at the lytic synapse is required for granule release, while actin recovery prevents prolonged secretion [93,94]. A positive correlation between actin dynamics and the concentration of PtdIns(4,5)P_2_ by phosphatidylinositol 4,5-biphosphate kinases (PIP5Ks) at the synaptic membrane has also been reported [95]. 

Actin dynamics is also required for TCR-MCs formed at the periphery to move to the center of the IS [96]. This centripetal actin flow is driven by actin polymerization in concert with MyoIIA [97]. Multiple signaling pathways triggered at the IS result in the activation of actin regulatory proteins, such as members of the Wiskott–Aldrych syndrome protein (WASP)/SCAR family (i.e., WAVE2 and WASP) that, in turn, activate the Arp2/3 complex to nucleate new actin filaments. Indeed, engagement of the TCR, the costimulatory molecule CD28 and the adhesion molecule LFA-1 all trigger signaling cascades that converge in the activation of Vav1, a guanine nucleotide exchange factor (GEF) critical for the actin regulators Rac1 and Cdc42 and their effectors WAVE2 and WASP, respectively [98]. WAVE2 is responsible for the formation of a branched actin network that is required for T cell spreading and regulation of integrin-dependent adhesion [99,100,101]. At variance, WASP generates small actin patches, known as “foci”, which colocalize with the TCR-MCs and are associated with local membrane protrusions that favor TCR:pMHC interactions [102,103]. Other important players in actin cytoskeleton remodeling at the IS are formins [104,105], which nucleate long actin filaments at the cell periphery that are organized into antiparallel concentric arcs by MyoIIA. Using structured-illumination microscopy, Murugesan et al. have observed a specific localization of active LFA-1 and TCR-MCs along and inside the arcs, respectively, which is important for maintaining IS symmetry and for T cell activation. Formin-dependent reorganization of the actin cytoskeleton is also involved in centrosome polarization to the IS [105].

Collectively, these findings support the notion that the actin cytoskeleton does not simply act as a scaffold for building the IS, but also participates in the regulation of TCR triggering and downstream signal transduction. Interestingly, the TCR itself was recently identified as a mechanosensor, sensing and responding to mechanical forces that are generated by cytoskeleton remodeling events at the contact area with the APC [106,107,108,109]. Mechanosensing is also an important function of the primary cilium, as demonstrated for polycystin-1 and -2 (PC-1 and -2), which interact to form a Ca^2+^-permeable nonselective cation at primary cilium of renal epithelial cells, where they are activated by the urine flow [110]. The mechanosensing function of the primary cilium is however mainly regulated by the stiffness of the axonemal microtubules [69,71]. This indicates the unique roles of the actin and microtubule cytoskeletons in mechanosensing at the IS and the primary cilium.

#### 3.1.5. Emerging Implications of Septins in the Assembly of Polarized Structures

Septins belong to a family of GTP-binding proteins that is highly conserved in eukaryotes and are now recognized as cytoskeletal proteins [111,112]. Although they are not major components of cilia, septins were found at the primary cilium, with a preferential localization at the transition zone or at the axoneme. From as yet sparse pieces of evidence, septins are able to interact with positive regulators of ciliogenesis, including Rab8 [113]. Moreover, microtubule associated protein 4 binds to septin 2 and competes with it for microtubule binding, thus regulating ciliary length [114]. Septins also organize a ring-like structure that was observed at the base of both primary cilia in IMCD3 cells and motile cilia in *Xenopus* embryos [115,116], where it controls ciliary length and function. 

Intriguingly, septins were also observed to assemble a ring around the IS. However, there are divergent opinions on the role of septins in T cell signaling. Mujal et al. did not observe defects in TCR-dependent calcium signaling in septin-deficient T cells [117], while others had previously described an impaired calcium flux caused by the mislocalization of the calcium release-activated calcium channel protein 1 and the stromal interaction molecule 1 in the absence of septins [118,119]. An interesting hypothesis to be tested could be whether the septin ring at the IS might function as a diffusion barrier that contributes to limit lateral membrane diffusion, similar to what occurs at the primary cilium.

### 3.2. Vesicular Trafficking to the Primary Cilium and the Immunological Synapse

#### 3.2.1. An Overview of Membrane-Associated Protein Trafficking to and into the Cilium 

Cilia biogenesis and function rely on the regulated transport of building blocks as well as receptors and signaling components in and out of the cilium. Targeting of membrane-associated proteins to the cilium is thought to be driven by specialized signal sequences, known as ciliary targeting sequences (CTSs). CTSs interact with components of the trafficking machinery and promote ciliary localization via tailor-made trafficking pathways. However, when ectopically expressed in ciliated cells, the T cell adaptor LAT is transported to the cilium despite the absence of a CTS [51], suggesting that, at least in specific cases, CTSs could be dispensable for ciliary targeting. 

From studies carried out on photoreceptor cells, it is known that sorting of rhodopsin is initiated at the Golgi apparatus, where the GTPase ARF4 binds to its CTS [120]. A set of regulators, including ARF4, the Arf GTPase-activating protein (GAP) ASAP-1, Rab11 and its effector FIP3, Rab8 and its GEF Rabin8 regulate the budding, transport to and fusion of rhodopsin-containing vesicles with the periciliary membrane [121]. Alternatively, ciliary receptors transit through recycling endosomes and then delivered to the periciliary compartment. In this pathway, several transport components, which are known to be associated with recycling endosomes, such as Rab8 and its GEF Rabin8, Rab11, Rab17 and its GAP TBC1D7, the Rab23-specific GAP EVI5like, the exocyst complex Sec10 and the tethering complex TRAPPII have been implicated in ciliogenesis [122,123,124,125,126]. An additional regulator of ciliary trafficking is IFT20, which participates in both direct and recycling trafficking pathways [127,128,129]. Other ciliary receptors, including Smoothened, exploit a third route by reaching the plasma membrane and then laterally moving to the ciliary membrane [130]. Another pathway is specific for N-myristoylated ciliary proteins that exploit the cargo adapter Unc119, the Arf-like GTPase ARL3 and its GAP RP2 to localize to the cilium [131]. For an extended list of ciliogenesis regulators the reader is referred to Table 1 and the references therein.

The transition from vesicular trafficking to the base of the cilium to the transport of ciliary proteins within the cilium requires the engagement of an IFT machinery that includes three multimolecular coat-like subcomplexes, known as IFT-A, IFT-B and the BBSome [165]. In a simplified picture, ciliary components are bidirectionally shuttled up and down the length of the cilium by IFT particles that couple with molecular motors to move along axonemal microtubules. IFT particles consist of a complex A and a complex B that move together, even though IFT-A is mainly involved in anterograde transport and IFT-B in retrograde transport. In this context, the BBSome has been proposed to cooperate with the IFT system in the transport of proteins implicated in signaling rather than structural components. In addition, the BBSome is a key player in the activation of Rab8 by Rabin8 [122,123,124]. Active Rab8 enters the primary cilium with the assistance of the IFT-A component IFT121 [166] and mediates the crossing of a diffusion barrier by ciliary cargo and their concomitant transport into the ciliary compartment. Recently, more specific functions have been identified for each complex. The IFT-A complex and the factor responsible for its recruitment to the membrane, TULP3, were found to be involved in the constitutive entry of GPCRs [167]. Consistent with its role in the Rabin8-dependent activation of Rab8, the BBSome plays a central role in ciliary import, as witnessed by the defective localization of ciliary receptors in cells depleted of Bardet Biedl syndrome (BBS) proteins or their regulators [168,169,170]. Nevertheless, the BBSome and the Arf-like GTPase ARL6 were also demonstrated to participate in the signal-dependent retrieval of receptors from the primary cilium [43,171], suggesting a complementary function for IFT-A and the BBSome in receptor trafficking. Hence the BBSome could be a regulator of both ciliary entry and exit, depending on the cargo and cell type. This raises the question whether the BBSome and the IFT-B complex have a redundant function. Several laboratories have started to address this question by investigating where the BBSome coat is assembled within the cell and which is the trafficking step regulated by this complex. Based on the fact that BBSome is able to polymerize a coat on phospholipid bilayers without inducing membrane deformation [146], one interesting hypothesis, that will need to be experimentally addressed, posits the BBSome as a coat adaptor for the IFT-B complex. In this scenario, the BBSome may help to initiate cargo clustering and to recruit the IFT-B complex to ciliary cargo, functioning similar to the adaptor AP-1 in the formation of clathrin-coated vesicles. The existence of a bilayered IFT-B/BBSome coat observed by super-resolution stochastic reconstruction microscopy [164,172] could represent the first evidence in support of this notion. 

#### 3.2.2. On the Way to the IS: Ciliary Regulators of Conserved Trafficking Machinery

**Figure 2:**
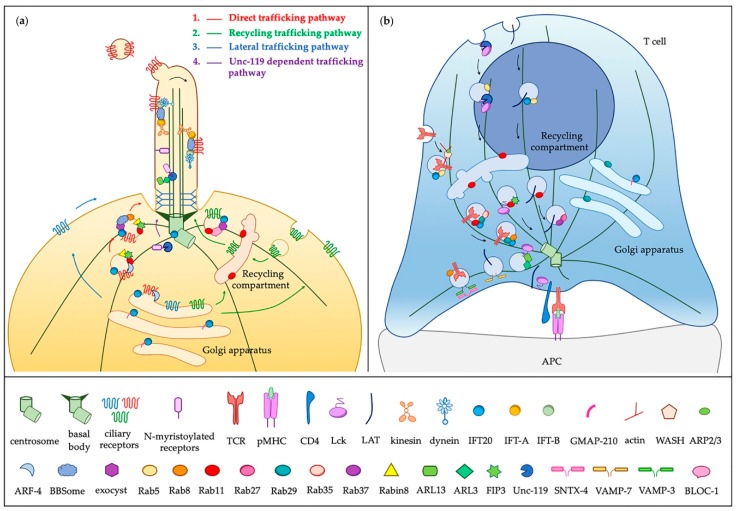
Vesicular trafficking at the primary cilium and the IS. The signaling function of both the primary cilium and the IS relies on the delivery of receptors and signaling mediators to a specialized membrane patch. (**a**) In ciliated cells membrane-associated proteins are sorted at the TGN into vesicles that reach the base of the cilium either directly (direct trafficking pathway, in blue) or through recycling endosomes (recycling trafficking pathway, in red), and then dock to the periciliary membrane. Alternatively, vesicles carrying ciliary receptors fuse with the plasma membrane and receptors are then transferred to the ciliary membrane by lateral diffusion (later trafficking pathway, in blue). A specific pathway for N-myristoylated proteins involves Unc119-RP2-ARL3 (Unc-119 dependent trafficking pathway, in purple). Within the cilium, the bidirectional transport of proteins depends on the IFT-A and IFT-B subcomplexes that move along the axoneme in association with molecular motors. The BBSome stabilizes the interaction between IFT-A and IFT-B during anterograde transport, while it helps the recruitment of receptors by IFT-B allowing for their retrograde transport. Activated receptors that are not retrieved back to the cell body undergo ectocytosis from the ciliary tip. (**b**) Polarized recycling together with passive lateral diffusion and active-mediating movement of TCR-microclusters (TCR-MCs) drive the accumulation of TCRs and signaling molecules at the IS. This process involves the microtubule-dependent polarized transport of intracellular pools associated with recycling endosomes. The translocation of the centrosome and associated Golgi apparatus as well as of the recycling compartment to a site just beneath the IS is a crucial event for the establishment of polarized vesicular trafficking. In addition to general recycling regulators (i.e., Rab4, Rab5 and Rab11), different Rab GTPases, IFT proteins, SNAREs and adapters are combined to specifically control the polarized transport of the TCR, LAT and Lck to the IS. From the dissection of these pathways, several proteins have emerged as shared participants in ciliogenesis and IS, suggesting that the non-ciliated T cells co-opt components of the ciliary machinery to control polarized recycling. Regulators, the function of which has not been mapped to a specific step in the pathways yet, are not depicted in the figure.

Consistent with their high level of structural homology with coatamers [173], the role of the IFT system in ciliated has recently been shown not to be limited to ciliogenesis. Two IFT proteins, IFT20 and IFT88, have been shown to mediate the starvation-induced transport of several components of the autophagic machinery, including the phagophore elongation complex component ATG16L1, to and into the primary cilium [174]. This evidence suggests that IFT proteins carry out extraciliary functions and act as general regulators of vesicular trafficking in ciliated cells. Interestingly, this function is conserved in cells lacking a primary cilium, namely T cells. Naïve T cells exploit vesicular trafficking to deliver recycling TCRs to the IS and sustain a long-lasting signaling required for T cell activation (Figure 2). In this context, we have identified components of the IFT system as unexpected players in recycling pathways of molecules that capitalize on this mechanism to accumulate at the IS. Namely, we have demonstrated that IFT20 promotes IS assembly by selectively controlling the polarized recycling of membrane receptors (i.e., the TCR/CD3 complex, the transferrin receptor) and of the membrane-associated adaptor protein LAT [21,33,175]. Further characterization of TCR recycling by our and other labs has resulted in the identification of an array of specific regulators involved in this pathway (Table 1 and references therein) that include IFT proteins (i.e., IFT-20, -52, -54, -57 and -88), IFT20-binding partners (i.e., ARPC3 and ERGIC-53), Rab GTPases (i.e., Rab3d, Rab8b, Rab29, Rab35 and its GAP EPI64C) and soluble NFS attachment protein receptor (SNARE) proteins (i.e.,VAMP-3, SNAP-23, syntaxin-4 and -17), beyond the general regulators of endosome recycling Rab5, Rab4 and Rab11 [176,177,178] (Figure 2).

In addition to the TCR, T cells repurpose molecules of the ciliary machinery to ensure the polarized traffic of signaling mediators (i.e., LAT and Lck) to the IS (Figure 2). For instance, VAMP-7, which is the unique vesicular (v) -SNARE implicated in ciliogenesis without a clear mechanism of action [162], controls the recruitment of LAT-containing vesicles to the IS [160]. Another example is the epithelial cell polarization and ciliogenesis regulator myelin and lymphocyte protein (MAL) [157] that has been demonstrated to control the correct sorting and targeting of Lck and LAT to distinct membrane subdomains of the IS [153]. Recently, Stephen et al. have demonstrated that, similar to ciliated cells, Unc119 and ARL3-ARL13B are required for the rapid mobilization of Lck to the IS. According to their results, myristoylated Lck is extracted from the membrane at sites distal from the synapse by Unc119 and then redirected to the IS, where active ARL3 promotes its local release [138]. Additionally, the Rab11 effector FIP3, which facilitates the interactions of ASAP1 and Rab11 with Rabin8 in early ciliogenesis [143], was found to orchestrate the delivery of Lck-containing vesicles as well as of the Rho-family GTPase Rac1 to the IS [134]. Regulators shared by the primary cilium and the IS appear to also act in the endocytic pathway of signaling molecules. This is exemplified by Rab6, which is required for the anterograde transport of vesicles containing PC-1 in ciliated cells [140], but has recently been shown to regulate the retrograde transport of LAT internalized at the IS to the TGN during its recycling [179].

Collectively, these findings provide evidence of vesicular traffic-related extraciliary functions of “ciliary” proteins (Figure 2). They also underscore the ability of non-ciliated T cells to co-opt components of the ciliary machinery for IS formation, a process that, similar to ciliogenesis, relies on vesicular trafficking. Moreover, both the primary cilium and the IS exploit spatially and temporally regulated protein trafficking to adapt the composition of the signaling compartment to signaling demands. This occurs through the tailoring of pre-existing trafficking modules and regulators to generate unique routes that control the delivery of specific receptors to specialized membrane patches. Customized pathways have been in part unraveled both in ciliated and non-ciliated T cells. For instance, within the recycling pathway regulated by Rab11, which is a general regulator of polarized recycling to the IS, IFT20 is a key player in the orchestration of the pathway that controls the delivery of different recycling receptors to the synaptic membrane. Based on our results, the Rab11-dependent pathway branches into a pathway involving IFT20 responsible for TCR and TfR traffic to the IS, and a separate, IFT20-independent pathway for CXCR4 [33]. In ciliated cells, a recent work by Monis et al. has shown that, similar to IFT25 and IFT27 [180,181,182], IFT20 is required for the trafficking of some ciliary receptors, but dispensable for others. A pathway mainly independent of IFT20 promotes the direct traffic of Smoothened to the plasma membrane, while an IFT20-dependent pathway, which involves also the exocyst subunits Exo70 and Sec8, controls the ciliary delivery of fibrocystin from the Golgi complex to the ciliary base. An additional endosomal pathway requires both IFT20 and the biogenesis of lysosome-related organelles complex (BLOC-1) to ensure PC-2 transit through the intermediate recycling compartment before reaching the primary cilium [129]. 

### 3.3. Breaking the Phospholipid Code 

Another common feature shared by the IS and the primary cilium is lipid specialization. Phosphoinositides (PIs) are differentially distributed in specific domains of the IS and the primary cilium, thereby establishing a phosphoinositide “code” that influences multiple processes, including signaling and cytoskeleton remodeling. The ciliary membrane is physically continuous with the plasma membrane, but differs in lipid composition. Phosphatidylinositol 4-phosphate (PtdIns4P) is the major component of the ciliary membrane, while PtdIns(4,5)P_2_ is transiently enriched at the transition zone and converted into phosphatidylinositol 3,4,5-triphosphate (PtdIns(3,4,5)P_3_) [183,184]. Although the role of PtdIns4P is still poorly investigated, depletion of the PtdIns4P-binding protein FAPP2 leads to a disruption in the transport of newly synthetized proteins to the primary cilium [185]. Conversely, the functional relevance of PtdIns(4,5)P_2_ is well-established, as witnessed by the correlation between an altered distribution of PtdIns(4,5)P_2_ at the primary cilium and signaling defects. This is consistent with the fact that the interaction between the IFT-A complex and PtdIns(4,5)P_2_, which is mediated by the tubby-like protein TULP3, promotes the ciliary transport of vesicles carrying negative regulators of Hh signaling (i.e., the orphan GPCR Gpr161) and other membrane associated proteins (e.g., ARL13B and INPP5) [167,186,187]. The concentration of PtdIns(4,5)P_2_ at the ciliary membrane is dynamically regulated by 5-phosphatases and kinases through the rapid conversion of one inositol phospholipid to another. In particular, the role of two 5-phosphatases, OCRL1 and INPP5E, have been extensively investigated in ciliogenesis, as mutations in their coding sequences are pathogenic in Lowe syndrome and Joubert syndrome, respectively [188,189]. Both these 5-phosphatases show a ciliary localization. Moreover, defects in their activity lead to an increased ciliary concentration of PtdIns(4,5)P_2_ with relevant implications in downstream signaling pathways. For instance, ciliary localization of Gpr16 enhances in the absence of INPP5 due to the retention of TULP3 and IFT-A [184], while a decreased accumulation of proteins involved in Hh signaling pathway was observed in mouse embryonic fibroblasts derived from a Lowe syndrome mouse model [190]. Thus, defects in inositol phosphate metabolism may help to explain the ciliary dysfunctions observed in multisystem disorders, including Lowe syndrome and Joubert syndrome. 

In T lymphocytes PIs have been involved in spatial regulation of signaling events, as PtdIns(4,5)P_2_ is a precursor of second messengers, such as inositol triphosphate (IP3) and DAG, on which signal transduction by the TCR and co-stimulatory receptors depends. PtdIns(4,5)P_2_ also serves as a substrate of class I PI3-kinase that controls the activation of the Akt pathway, a central regulator of the development, maturation and function of immune cells by generating PtdIns(3,4,5)P_3_. An inverse correlation between the phosphorylation status of PtdIns(4,5)P_2_ and the cytoplasmic domain of the CD3ε chain was found in a mouse T-cell hybridoma [191], suggesting that PIs may act earlier in the pathway by controlling TCR/CD3 complex dynamics and its activation at the plasma membrane. After TCR triggering, an increase in the concentration of DAG is generated through the cleavage of PtdIns(4,5)P_2_ by phospholipase Cγ1. This event leads to changes in the lipid composition at the IS, where molecules involved in lipid metabolism are displaced from the membrane as a consequence of the general rise in membrane charge. This is the case of PIP5Ks that electrostatically bind PtdIns(4,5)P_2_ itself [192,193] and are released following TCR activation [95]. The pharmacological inhibition of PIP5Kα by ISA-2011B was observed to weaken CD28 signals and to induce an upregulation of Th17-related inflammatory cytokines in T lymphocytes from type 1 diabetes patients [194]. Differently, the recruitment of PIP5K betaβ to the IS is important for the synaptic accumulation of filamin A and lipid rafts [195].

A further level of complexity is added by the fact that phosphoinositide signals are important for the coordination of cytoskeleton rearrangements. In ciliated cells PtdIns(4,5)P_2_ activates multiple regulatory proteins of the actin cytoskeleton (e.g., WASP family members, cofilin and gelsolin) promoting actin polymerization [196,197,198,199]. In T cells PtdIns(4,5)P_2_ is able to promote the polymerization of cortical actin at the IS as well by interacting with a number of actin-related proteins, including Exrin/Radaxin/Moesin and WASP [200,201]. Moreover, a study carried out on CD4^+^ and CD8^+^ T cells has demonstrated that the diacylglycerol kinase α is required for the establishment of a DAG gradient across the IS, with the maximal accumulation at the cSMAC that drives centrosome polarization [202]. Recently, a prolonged accumulation of PtdIns(4,5)P_2_ across the synapse, which is induced by either treatment with a phospholipase Cγ inhibitor or blocking the dissociation of PIP5K proteins from the plasma membrane, has been documented to prevent actin clearance from the cSMAC, thus impairing both centrosome docking and granule secretion in CTLs [95]. Hence a dynamic balance between the production and breakdown of PIs enables the cell to respond to extracellular cues by modulating the signaling function of the primary cilium and the IS, in terms of receptor localization, changes in second messenger production and cytoskeleton dynamics. 

### 3.4. Front-to-Rear Polarization during Early Ciliogenesis and IS Assembly

Given that the IS and the primary cilium are polarized structures, it is not surprising that polarity proteins redistribute in the cell during IS assembly and ciliogenesis. Components of the partitioning-defective (Par) and Crumbs complexes, such Par3, Par6 and Crb3, and other polarity-related proteins, including segment polarity protein disheveled homolog and atypical protein kinase C-ζ (PKC-ζ), all localize at the primary cilium contributing to the correct positioning of the basal body [203,204]. Many of these proteins have now been shown to be involved in T cell polarity during migration and stabilization of a polarized contact with the APC. Par6 and aPKC-ζ have been implicated in the motility toward and scanning of the APC by T cells [205]. Following encounter of a cognate APC, IS assembly involves the accumulation of Par3 and the active form of aPKC-ζ at APC-T cell interface, while the mammalian homologues of the *Drosophila* Scribble and Discs large localize to the distal pole of the cell [206,207]. Surprisingly, several hours after stimulation, this distribution pattern undergoes an inversion with Discs large localizing to the IS, where it controls microtubule organization and CD4^+^ T cell activation by acting as a scaffold protein for the actin-cytoskeleton linker protein ezrin [63]. Conversely, PKC-ζ and PKC-λ/ι move to the distal pole to control the asymmetric distribution of cell fate determinants during the first division of CD8^+^ T cells [208,209,210,211], with the two daughter cells differentiating into an effector and a memory cell. Interestingly, polarity-related proteins have also been implicated in CD4^+^ T cell differentiation, as exemplified by PKC-ζ and PKC-λ/ι for Th2 cells [212,213] and adenomatous polyposis coli for regulatory T cells [214]. 

## 4. Investigating Extraciliary Functions of Ciliary Proteins Has Opened New Scenarios 

The similarities in the architectural framework of the primary cilium and the IS [215], taken together with the fact that a variety of ciliary proteins are co-opted by T cells for IS assembly, support the notion that the primary cilium and the IS are homologous structures. It is noteworthy that the majority of these proteins operate in vesicular trafficking. This suggests the existence of a highly conserved traffic machinery that is exploited for cilium-independent functions both in ciliated and non-ciliated cells. This notion has been underscored in ciliated cells with the implication of the IFT system in cell autophagy and collagen trafficking in neural crest cells [174,216] and further extended to the non-ciliated T cells, beyond IS assembly. In fact, starting from the observation of a defect in autophagic clearance and an accumulation of lipid droplets in IFT20-deficient T cells, we have recently demonstrated that IFT20, which controls cargo trafficking to the cilium and the IS, contributes to the delivery of acid hydrolases to the lysosome by controlling retrograde transport of the recycling cation-independent mannose-6-phosphate receptor to the TGN [217]. This lysosome-related function of IFT20 is shared by non-ciliated and ciliated cells [217].

While an expanding array of ciliary proteins is being implicated in extraciliary functions, reciprocally proteins that operate in cilia-unrelated cellular pathways have been shown to participate in ciliogenesis and cilia function. For instance, transcriptional factors belonging to the regulatory factor X family are required for the activation of a ciliogenic programme and cooperate with the transcription factor forkhead box protein J1 in the formation of specialized cilia [218]. Moreover LKB1, a key kinase in the AMPK and mTOR pathway, was found to interact with the receptor PC-1 and the ciliary protein NPHP1 at the primary cilium, where it regulates metabolic signaling as well as a cilium-induced expression of pro-inflammatory chemokines (i.e., CCL2) in renal epithelial cells [219]. 

Finally, the investigation of ciliogenesis-related proteins may lead to a rapid progress in our understanding not only of the complex ciliopathy phenotypes, but also of immune disorders characterized by defective T cell-mediated responses. Interestingly, mutations in the gene encoding Unc119, which has a well-established role in ciliogenesis, have been associated with impaired TCR signaling in idiopathic CD4 lymphopenia [220]. Hence the homology between the primary cilium and the IS provides a unique resource for elucidating mechanisms with relevant implications in both physiological and pathological contexts. 

## Figures and Tables

**Table 1:** An expanding array of vesicular trafficking regulators in IS assembly and ciliogenesis.

Function	Immunological Synapse	Primary Cilium
GTPases	Rab3 [132]; Rab4 [33]; Rab5 [33]; Rab8 [132,133]; Rab11 [33,132]-FIP3 [134,135]; Rab27 [132]; Rab29 [136]; Rab35 [137]; Rab37 [132]; ARL3 [138]	Rab5 [139]; Rab6 [121,140]; Rab8 [123]; Rab10 [141]; Rab11 [124,142]-FIP3 [143]; Rab17 [125]; Rab23 [144]; Rab29 [136]; ARF4 [120]; ARL3 [131,145]; ARL6 [146]
GEFs and GAPs	EPI64C [137]; ARL13B [138]	RP2 [145,147]; Rabaptin5 [148]; ASAP-1 [121]; Rabin8 [123]; TBC1D7 [125]; EVI5like [125]; ARL13 [149,150]
Adaptors	WASH [151,152]; MAL [132,153]; clathrin [154]; β-arrestin1 [155]; EB-1 [48]; Unc119 [138]; ARPC3 [22]	TRAPPII [124]; AP-1 [156]; AP-2 [139]; MAL [157]; EB-1 [67,158]; Unc119 [131]
SNAREs and tethers	SNAP-23 [32]; Syntaxin-4 and -17 [32,159]; VAMP-2, -3 and -7 [32,132,133,160]	Exocyst subunits (Sec10, Exo70) [126,129], Syntaxin-10 [161]; VAMP-7 [162]
Others	IFT20; IFT52; IFT54; IFT57; IFT88 [21,33]; ERGIC-53 [22]	IFT proteins [163]; BLOC-1 [129]; BBSome complex [164]

Regulators involved in the formation of the IS and the primary cilium include GTPases, guanine nucleotide exchange factors (GEFs) and GTPase-activating proteins (GAPs), adaptors, soluble NFS attachment protein receptor (SNARE) proteins and tethering molecules that are listed in the table. Some molecules have been identified as specific regulators of IS assembly or ciliogenesis since their function in the formation of the homologue structure has not been assessed yet (in black). Others are now known as shared participants in the assembly of the IS and the primary cilium (in red).
